# Supplementary material for: Trioleyl Pyridinium, a Cationic Transfection Agent for the Lipofection of Therapeutic Oligonucleotides into Mammalian Cells
Source: Pharmaceutics. 2023 Jan 27;15(2):420. doi: 10.3390/pharmaceutics15020420 (PMC9960667; doi:10.3390/pharmaceutics15020420)
Supplement: Supplementary file 1 [file pharmaceutics-15-00420-s001.zip › pharmaceutics-2170280-supplementary.pdf]

pharmaceutics

MDPI

Supplementary Materials: Trioleyl Pyridinium, a Cationic Transfection Agent for the Lipofection of Therapeutic Oligonucleotides into Mammalian Cells

Ana Delgado, Rosa Griera, Núria Llor, Ester López-Aguilar, Maria Antònia Busquets, Véronique Noé and Carlos J. Ciudad *

A

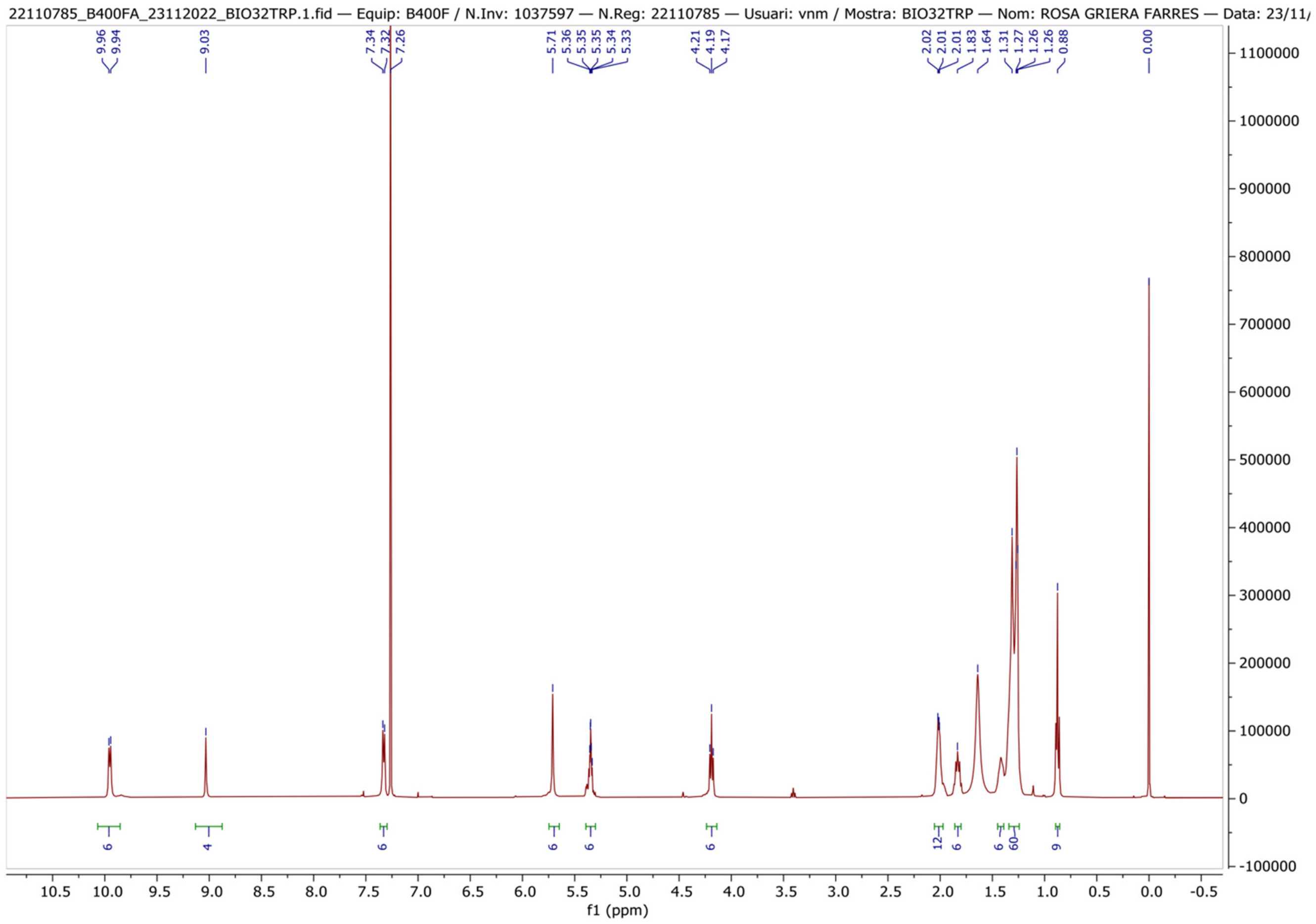

B

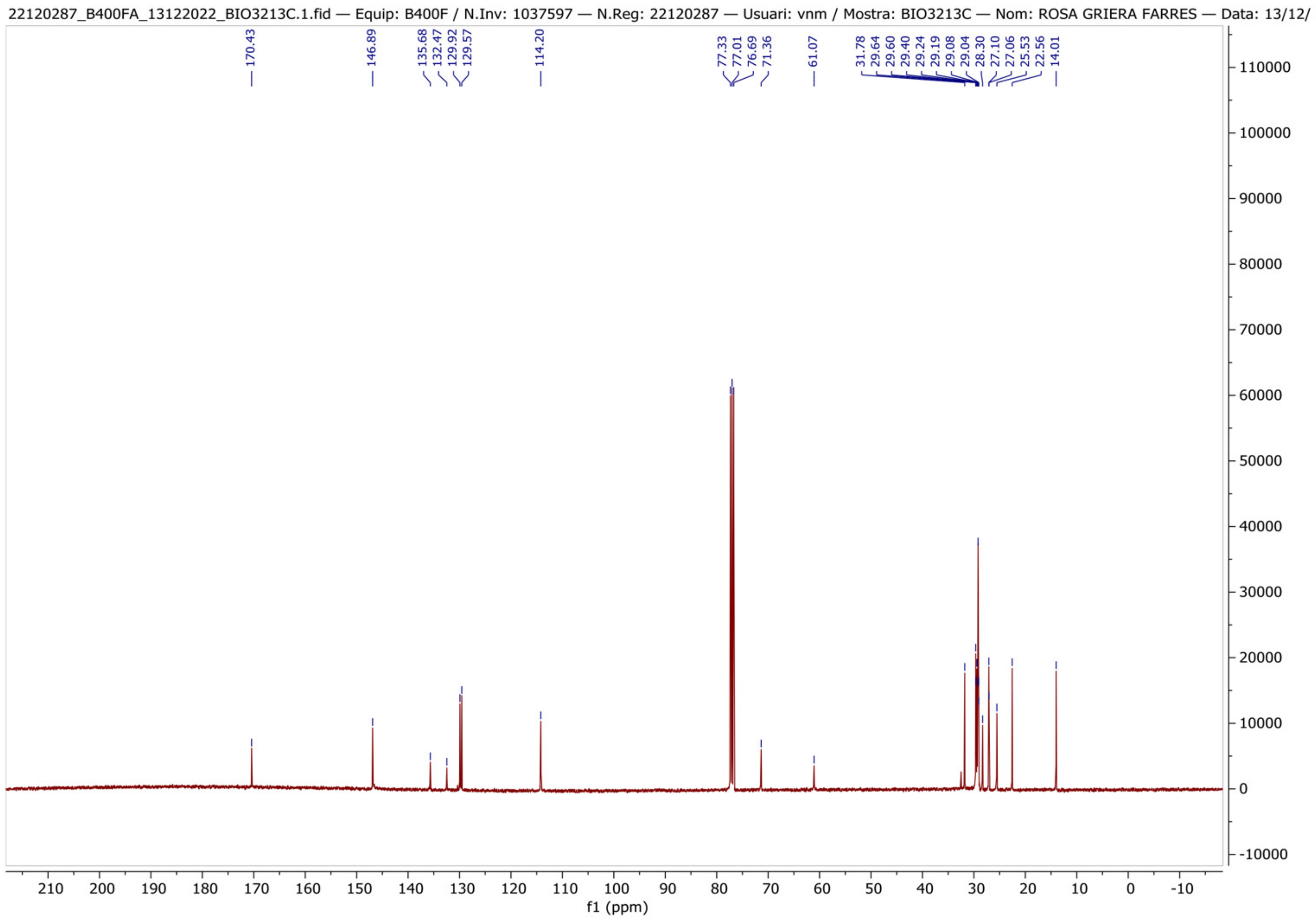

Figure S1. ^{1}H NMR (**A**) and ^{13}C NMR (**B**) spectra of TROPY.
